# Longitudinal Assessment of Creatine Kinase, Creatine/Creatinine_ratio_, and Myostatin as Monitoring Biomarkers in Becker Muscular Dystrophy

**DOI:** 10.1212/WNL.0000000000201609

**Published:** 2023-02-28

**Authors:** Nienke M. van de Velde, Zaïda Koeks, Mirko Signorelli, Nisha Verwey, Maurice Overzier, Jaap A. Bakker, Gautam Sajeev, James Signorovitch, Valeria Ricotti, Jan Verschuuren, Kristy Brown, Pietro Spitali, Erik H. Niks

**Affiliations:** From the Departments of Neurology (N.M.V., Z.K., J.V., E.H.N.), Biomedical Data Sciences (M.S.), Human Genetics (N.V., M.O., P.S.), and Clinical Chemistry and Laboratory Medicine (J.A.B.), Leiden University Medical Center, the Netherlands; Duchenne Center Netherlands (N.M.V., J.V., P.S., E.H.N.); European Reference Network for Rare Neuromuscular Diseases [ERN EURO-NMD] (N.M.V., Z.K., N.V., M.O., J.V., P.S., E.H.N.); Mathematical Institute (M.S.), Leiden University, the Netherlands; Analysis Group Inc (G.S., J.S.), Boston, MA; Solid Biosciences Inc (V.R., K.B.), Cambridge, MA; and NIHR Great Ormond Street Hospital Biomedical Research Centre (V.R.), Great Ormond Street Institute of Child Health, University College London, & Great Ormond Street Hospital Trust, United Kingdom.

## Abstract

**Background and Objectives:**

The slow and variable disease progression of Becker muscular dystrophy (BMD) urges the development of biomarkers to facilitate clinical trials. We explored changes in 3 muscle-enriched biomarkers in serum of patients with BMD over 4-year time and studied associations with disease severity, disease progression, and dystrophin levels in BMD.

**Methods:**

We quantitatively measured creatine kinase (CK) using the International Federation of Clinical Chemistry reference method, creatine/creatinine_ratio_ (Cr/Crn) using liquid chromatography–tandem mass spectrometry, and myostatin with ELISA in serum and assessed functional performance using the North Star Ambulatory Assessment (NSAA), 10-meter run velocity (TMRv), 6-Minute Walking Test (6MWT), and forced vital capacity in a 4-year prospective natural history study. Dystrophin levels were quantified in the tibialis anterior muscle using capillary Western immunoassay. The correlation between biomarkers, age, functional performance, mean annual change, and prediction of concurrent functional performance was analyzed using linear mixed models.

**Results:**

Thirty-four patients with 106 visits were included. Eight patients were nonambulant at baseline. Cr/Crn and myostatin were highly patient specific (intraclass correlation coefficient for both = 0.960). Cr/Crn was strongly negatively correlated, whereas myostatin was strongly positively correlated with the NSAA, TMRv, and 6MWT (Cr/Crn rho = −0.869 to −0.801 and myostatin rho = 0.792 to 0.842, all *p* < 0.001). CK showed a negative association with age (*p* = 0.0002) but was not associated with patients' performance. Cr/Crn and myostatin correlated moderately with the average annual change of the 6MWT (rho = −0.532 and 0.555, *p* = 0.02). Dystrophin levels did not correlate with the selected biomarkers nor with performance. Cr/Crn, myostatin, and age could explain up to 75% of the variance of concurrent functional performance of the NSAA, TMRv, and 6MWT.

**Discussion:**

Both Cr/Crn and myostatin could potentially serve as monitoring biomarkers in BMD, as higher Cr/Crn and lower myostatin were associated with lower motor performance and predictive of concurrent functional performance when combined with age. Future studies are needed to more precisely determine the context of use of these biomarkers.

Becker muscular dystrophy (BMD) is characterized by progressive muscle weakness caused by reduced levels of dystrophin with abnormal molecular weight.^1^ The conduction of clinical trials has been challenging in BMD because the low incidence (around 1:18,000 male live births, which is one-third of Duchenne muscular dystrophy [DMD]) severely hampers patient recruitment.^2^ Also, high functional variability and slow disease progression lead to difficulties capturing potential drug efficacy during a trial.^3^ Therefore, objective biomarkers are needed that could facilitate trial design and conduction.^4^ For example, prognostic biomarkers could enrich trials by selecting patients with a greater likelihood of having a clinical event, thereby allowing to reduce the sample size as recently outlined.^5^

Circulating biomarkers are of interest because of easy accessibility, limited patient burden, and relatively low costs. They may also provide a representation of the overall condition of patients. To identify possible blood-derived biomarkers in DMD and BMD, analyses have been performed through several methods including large-scale exploratory “-omics” approaches and more targeted approaches, for example, investigation of a limited number of molecules based on hypotheses or previous research.^6-11^ Markers related to muscle structure or integrity are of specific interest because of the replacement of muscle tissue by fat, a prominent sign of pathology in a muscle-wasting condition such as BMD. Creatine kinase (CK), commonly used as a diagnostic biomarker in muscular dystrophies, may not be optimal to monitor the disease due to the fact that its serum levels change because of both the membrane pathology and the amount of remaining muscle mass, next to other factors such as seasonal variation and dependence on physical activity.^12^ Alternatively, creatine, derived from diet or synthesized in the liver, is nonenzymatically converted to creatinine in muscle at a constant rate. Therefore, the conversion of creatine to creatinine is more closely related to the amount of preserved muscle tissue where this conversion takes place. Similarly, myostatin relates to muscle mass because it is produced and released by skeletal muscle tissue. It also acts as an inhibitor of excessive muscle growth.^13^ Indeed, creatine/creatinine_ratio_ levels were increased in patients with DMD, whereas serum myostatin was lower in BMD and DMD compared with healthy controls.^14-18^ Although these cross-sectional studies provide evidence that creatine/creatinine_ratio_ (Cr/Crn) and myostatin may be suitable as biomarkers, their relation to functional performance over a longer period still has to be largely demonstrated. Such data are important to more closely define the potential context of the use of circulating biomarkers.

We quantified CK, Cr/Crn, and myostatin as biomarkers in a longitudinal natural history study of adult patients with BMD and assessed correlations with disease severity, disease progression, and the ability to predict concurrent performance.

## Methods

### Patient Characteristics and Study Protocol

Participants were recruited from the Dutch Dystrophinopathy Database in a 4-year prospective BMD natural history study conducted at the Leiden University Medical Center between 2014 and 2019.^19^ Male subjects of age ≥18 years diagnosed with BMD based on the following criteria were included in the study: genetic confirmation (in-frame genetic variant) or another genetic variant in the *DMD* gene with a mild clinical phenotype (ambulant >16 years without steroid treatment). The study protocol consisted of 4 yearly 1-day visits with venous blood sampling and measurement of several functional tests including the North Star Ambulatory Assessment (NSAA), 10-meter walk/run test velocity (TMRv), the 6-Minute Walking Test (6MWT), and pulmonary function (forced vital capacity percentage predicted [FVC%]). Functional tests were performed by 2 trained observers as previously described.^20,21^ The NSAA was also performed in patients who lost ambulation during the study to capture decline of function in this scale. These patients could only score points on neck flexion (0–2 points) or sit from supine (0–2 points). Patients could also consent for a biopsy from the tibialis anterior (TA) muscle, taken at baseline or year 1 of the study.

### Standard Protocol Approvals, Registrations, and Patient Consents

The clinical data and human tissue have been obtained, stored, and handled in strict accordance with relevant guidelines and regulations. The study was approved by the Medical Ethical Committee of the LUMC. Written informed consent was obtained from all participants.

### Blood Sampling and Analysis

Blood samples were allowed to clot for 0.5–2 hours at room temperature in serum collection tubes (SST, 3.5 mL). Samples were further centrifuged for 10 minutes at 20°C at 2350 relative centrifugal force. Aliquots of 1.5 mL of serum were then frozen at −80°C pending use. Analysis of creatine and creatinine was performed using the method described previously.^22^ In brief, serum samples were mixed with the internal standard solutions (d3-creatine and d3-creatinine). Samples were deproteinized with acetonitrile. Supernatants were dried under nitrogen and derivatized with a mixture of butanol/acetyl chloride. Creatine was converted to its butyl ester, whereas creatinine remains underivatized, both were measured in micromoles per liter. Samples were dried again under nitrogen and reconstituted in the mobile phase. The compounds were separated using a Symmetry C18 column and detected in the MRM mode using tandem mass spectrometry.

Analysis of CK (U/L) was performed by colorimetric (International Federation of Clinical Chemistry) method on the Roche Cobas 8000. Analysis of myostatin (pg/mL) was performed by ELISA using a kit (cat. n. DGDF80) including the activation kit (cat. n. DY010) and quality control set 794 (cat. n. QC98). All products were obtained from R&D Systems, Bio-Techne, Minneapolis, MN, United States, and procedures were performed according to the manufacturer's instructions. Calculation of the myostatin values was performed using a Four-Parameter Logistic (4PL) Regression.

All analyses were performed in duplicates. The average of the samples was used in the analysis. Samples were excluded when the coefficient of variation (CV) exceeded 30%.

#### Dystrophin Quantification

Dystrophin percentage was quantified from available muscle biopsies of the TA muscle using capillary Western immunoassay as previously described.^23^ In 13 patients, a biopsy was available at baseline. In 7 other patients, muscle biopsies had been performed 4–5 years before baseline as part of a previous natural history study, yielding a total of 20 patients with dystrophin quantification.^24^

### Statistical Analysis

The longitudinal trajectories of Cr/Crn, CK, and myostatin were modeled using a linear mixed model^25^ for the log-transformed value of the biomarker, where we included a patient-specific random intercept and age as fixed-effect covariates. Patient-specific random slopes were not added because there were too few patients and data points available. The models were estimated using restricted maximum likelihood. The level of heterogeneity of each biomarker across patients was studied using the intraclass correlation coefficient (ICC). In a linear mixed model with random intercept, the ICC is an estimate of the correlation between repeated measurements from the same patient, and it provides an indication of how well the trajectories from different patients are separated. The association of the biomarkers with age was tested using an F test, with degrees of freedom determined using Satterwhite's correction.^26^ Multiple testing corrections were implemented using the Benjamini-Hochberg method for all the performed analyses.^27^

For patients with at least 2 measurements of a given functional test, the average yearly change of the functional test was estimated by fitting a linear regression model, with the functional test as response and time (in years) as covariate to all data points available for that patient.

Correlations of biomarkers with disease severity, disease progression, and dystrophin percentage across patients were assessed by measuring the Pearson correlation between the patient-specific random intercepts of the biomarkers and those of the functional tests. Random intercepts are then interpreted as the deviation of each patient from the mean of the studied cohort.

The prediction of concurrent performance was calculated using linear models with different combinations of predictors (age, Cr/Crn, CK, myostatin, and dystrophin). Predictive performance was measured with an optimism-corrected R^2^ based on the bootstrap.^28^

### Data Availability

Anonymized data can be made available to qualified investigators on request.

## Results

Thirty-six patients were included in the natural history study. One patient did not consent for blood sampling, and another patient was excluded because of withdrawal of consent after the baseline visit. The remaining 34 patients had a total of 123 visits. Serum samples were missing from 17 visits of 13 patients. CK levels were not analyzed at the fourth-year follow-up visit for 19 patients because no association with functional tests was found in the preliminary analysis of the first 3 study years. In 7 more visits, myostatin levels could not be determined (in 4 visits, not enough sample was left, and in 3 visits, the CV% was too high). Thus, Cr/Crn, myostatin, and CK levels were available at 106, 99, and 87 visits, respectively.

Baseline characteristics of patients are shown in Table 1. Eight patients were nonambulant (defined as an inability to perform the TMR without support) at baseline. Three patients lost ambulation during the study. The functional ability of the patients varied widely. For example, among the ambulant patients, the NSAA score at baseline ranged from 5 to 34 points. The FVC was above 80% in 26 of the 34 patients. Two patients were on nocturnal ventilatory support.

**Table 1:**
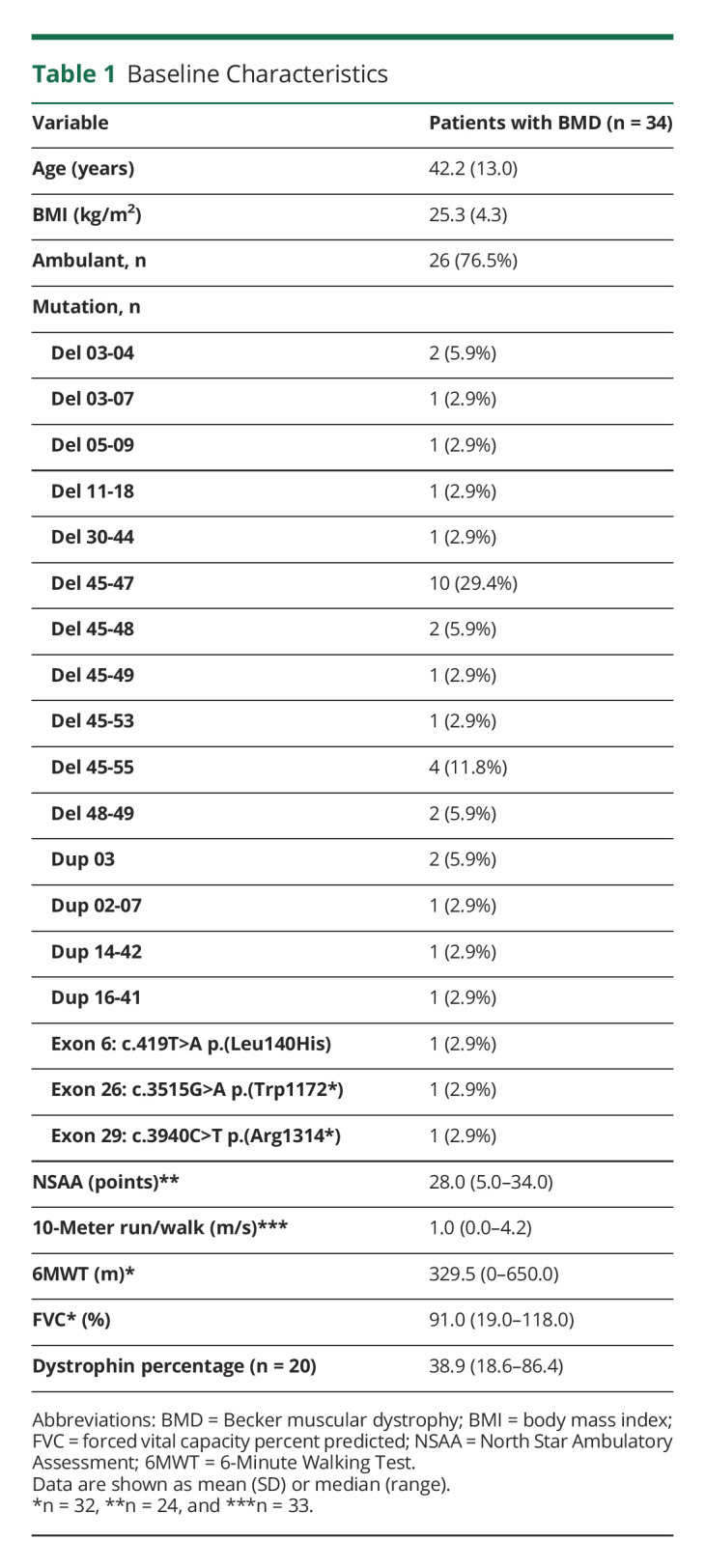
Baseline Characteristics

### Association of Biomarkers With Age and Cross-Correlation Across Biomarkers

**Figure 1:**
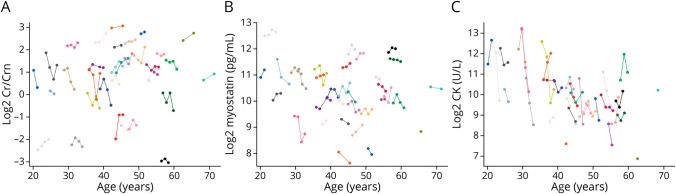
Change Over Time Biomarkers in Patients With BMD Creatine/creatinine_ratio_ (A), myostatin (B), and creatine kinase (C). Creatine/creatinine_ratio_ and myostatin were highly patient specific. Only creatine kinase declined significantly over time (adjusted *p* = 0.002). BMD = Becker muscular dystrophy; Cr/Crn = creatine/creatinine_ratio_; CK = creatine kinase.

**Figure 2:**
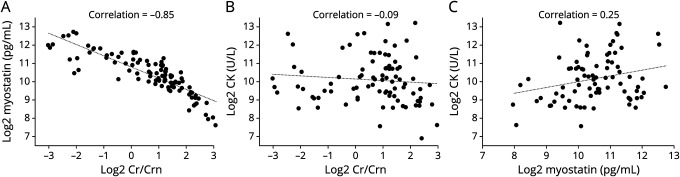
Cross-Correlation of Biomarkers in Patients With BMD Creatine/creatinine_ratio_ (A), myostatin (B), and creatine kinase (C). There was a high cross-correlation between creatine/creatinine_ratio_ and myostatin (A), but not with CK (B, C). BMD = Becker muscular dystrophy; Cr/Crn = creatine/creatinine_ratio_; CK = creatine kinase.

The ICC of Cr/Crn and myostatin was both high 0.960 (Figure 1A and B), indicating that the values of these 2 markers are strongly patient specific and highly heterogeneous across patients. Both biomarkers were not associated with age (adjusted *p* values = 0.626 and 0.147, respectively). CK was less patient specific (ICC = 0.550, Figure 1C) and declined with increasing age (adjusted *p* = 0.0002). The estimates from the mixed model for all the biomarkers are outlined in eTable 1 (links.lww.com/WNL/C505). The values of Cr/Crn and myostatin correlated highly with each other (ρ = −0.85, adjusted *p* < 0.001, Figure 2A) and showed low correlations with CK (adjusted *p* = 0.362 and *p* = 0.034, respectively, Figure 2B and C).

### Correlation of Biomarkers With Disease Severity, Disease Progression, and Dystrophin Percentage

**Figure 3:**
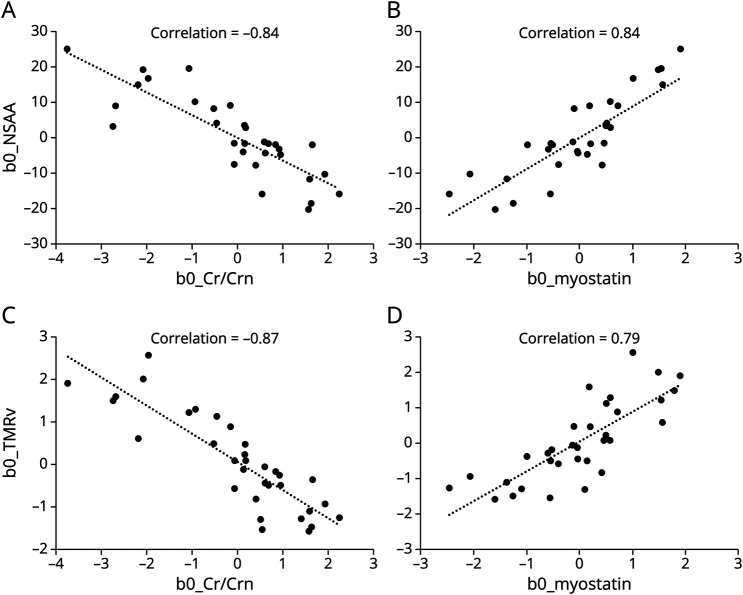
Correlation of Biomarkers With Functional Tests Across Patients The random intercepts of the North Star Ambulatory Assessment (A, B) and 10-meter run velocity (C, D) correlated highly with the random intercepts of creatine/creatinine_ratio_ and myostatin. Cr/Crn = creatine/creatinine_ratio_; NSAA = North Star Ambulatory Assessment; TMRv = 10-meter run velocity.

In ambulant patients, the NSAA, TMRv, and 6MWT showed strong negative correlations with Cr/Crn (ρ < −0.80, adjusted *p* < 0.001, Figure 3A and C and eFigure 1, links.lww.com/WNL/C505) and strong positive correlations with myostatin (ρ > 0.79, adjusted *p* < 0.001, Figure 3B and D and eFigure 1). As the NSAA was also performed in patients who lost ambulation during the study, we also correlated the biomarkers with the NSAA scores of only ambulant patients. Cr/Crn and myostatin were still highly correlated with the NSAA (ρ = −0.83 and ρ = 0.85, respectively, adjusted *p* < 0.001). The functional tests did not correlate with CK (ρ ≤ 0.25, adjusted *p* > 0.33, eFigure 1). The FVC correlated moderately with Cr/Crn (ρ = −0.45, adjusted *p* = 0.02), myostatin (ρ = 0.58, adjusted *p* = 0.001), and CK (ρ = 0.55, adjusted *p* = 0.003).

**Figure 4:**
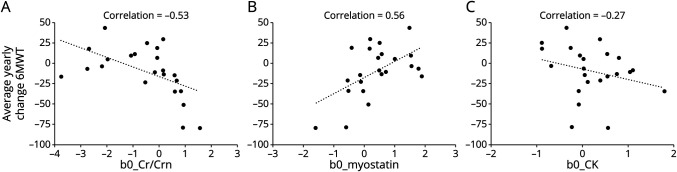
Correlation of Biomarkers With the Average Yearly Change of the 6MWT in Patients With BMD Average yearly change of the 6MWT correlated moderately with creatine/creatinine_ratio_ (A) and myostatin (B), and the correlation with creatine kinase (C) was weak. 6MWT = 6-Minute Walking Test; BMD = Becker muscular dystrophy; Cr/Crn = creatine/creatinine_ratio_; CK = creatine kinase.

Cr/Crn and myostatin correlated moderately with the average yearly change of the 6MWT (ρ = −0.53, adjusted *p* = 0.02 and ρ = 0.56, adjusted *p* = 0.016, respectively, Figure 4, A–C). All other correlations between the 3 biomarkers and the average yearly change of functional tests and pulmonary function were weak (−0.27 < ρ < 0.10, adjusted *p* > 0.39, eFigure 2, A–I, links.lww.com/WNL/C505). None of the 3 biomarkers correlated with dystrophin levels in muscle, quantified in the TA muscle (ρ < 0.17, eFigure 3, A–C).

### Prediction of Concurrent Functional Performance Using Age, Biomarkers, and Dystrophin Percentage

**Figure 5:**
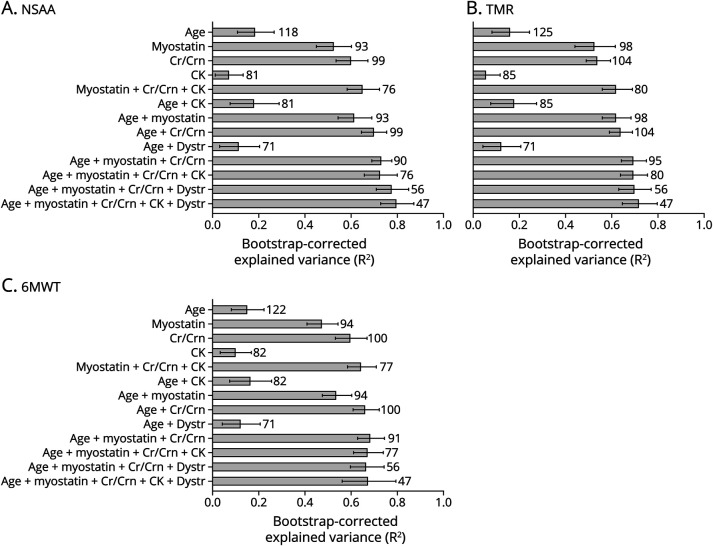
Prediction of Concurrent Functional Performance Using Age, Dystrophin, and Biomarkers North Star Ambulatory Assessment (A), 10-meter run test (B), and 6-Minute Walking Test (C). Numbers behind the bars indicate the amount of available data points for each prediction. Cr/Crn = creatine/creatinine_ratio_; CK = creatine kinase; dystr = dystrophin; NSAA = North Star Ambulatory Assessment; TMR = 10-meter run test; 6MWT = 6-Minute Walking Test.

The explained variance of concurrent performance, as assessed by the NSAA, TMRv, and 6MWT, using each predictor separately was highest using myostatin (bootstrap-corrected R^2^ = ∼50%) and creatinine/creatinine_ratio_ (bootstrap-corrected R^2^ = 55%-60%) (Figure 5). A combination of age and these 2 biomarkers increased the explained variance to about 75%. The dystrophin percentage did not seem to improve the prediction, although only 20 patients had dystrophin data available. Concurrent pulmonary function (FVC%) could be predicted around 35% by creatinine/creatinine_ratio_, 10% by myostatin, and 30% by the biomarkers combined with age (data not shown).

## Discussion

In this prospective 4-year study, we aimed to investigate whether Cr/Crn, myostatin, and CK could be biomarkers of disease severity and progression in BMD. Cr/Crn and myostatin, but not CK, were highly patient specific and correlated with disease severity but not with disease progression over 4 years. The explained variation of concurrent functional performance by these biomarkers together with age was up to 75%. Our study demonstrates that Cr/Crn and myostatin may be candidate monitoring biomarkers in BMD.

The lack of outcome measures capable of showing changes within the duration of a trial in rare and slowly progressive diseases has hindered the development and evaluation of potential drugs in clinical trials. Monitoring biomarkers have been defined by the Food and Drug Administration as a biomarker that is measured over time.^29^ Monitoring biomarkers in general may serve multiple purposes, including detecting the therapeutic effect or disease progression or determining how a drug is metabolized. Therefore, this term covers several types of biomarkers, including prognostic, predictive, response (pharmacodynamic), and safety biomarkers. Creatine used in the creatine/creatinine_ratio_ is derived from diet but can also be synthesized in the liver using guanidinoacetic acid and S-adenosyl-methionine. It functions as an energy buffer in high-energy demanding tissues such as skeletal muscle tissue. In periods of low muscle activity, creatine is phosphorylated to create phosphocreatine by the transfer of a phosphate group from adenosine triphosphate (ATP) to creatine. This reversible transfer is catalyzed by CK and yields adenosine diphosphate (ADP) and a phosphate group. In periods of high-energy demand in muscle cells, the phosphor group of phosphocreatine is transferred from ADP to create ATP and can occur within seconds of intense muscular effort.^30^ Creatine is nonenzymatically converted to creatinine in human muscle tissue in a constant rate of about 1.7% per day and is then released into the blood and cleared by the kidneys.^31^ With loss of muscle mass that is typical for muscular dystrophies, less creatine is converted into creatinine, leading to an increased ratio of creatine to creatinine. Serum creatinine has been shown to be reduced in several neuromuscular diseases including DMD, BMD, and spinal muscular atrophy.^14,15,32-34^ Two studies in a large cohort of mainly pediatric patients with dystrophinopathy demonstrated that creatinine levels distinguished DMD from BMD, and that it was inversely correlated with function; creatinine was also associated with disease progression in a smaller longitudinal subset with deteriorating DMD (n = 32) and BMD (n = 4) patients, with 2 time points spanning multiple years.^32,33^ Myostatin (or growth differentiation factor 8) is a member of the transforming growth factor β superfamily and acts as a muscle growth inhibitor.^35^ It is produced and released by myocytes and fibroblast residing in skeletal muscle tissue.^36^ Myostatin circulates in a latent form in the blood as a complex of 2 C-terminal dimers and 2 N-terminal inhibitory prodomains. When the prodomains are cleaved, the myostatin dimer becomes active and can bind to its receptor complex activin receptor type IIB (ActRIIB) and activin receptor–like kinase (ALK) 4 or ALK5 type 1 receptor in muscle tissue. On binding, myostatin inhibits muscle growth in 3 different pathways; myogenesis is downregulated through mitogen-activated protein kinase activation and through Smad 2/3 phosphorylation and subsequent nuclear translocation, and protein synthesis is reduced by the inhibition of mammalian target of rapamycin signaling.^37^ Inhibition of the myostatin pathway has been shown to lead to muscle hypertrophy in mice models for various muscular dystrophies.^38-41^ Circulating myostatin levels seemed to be lower in patients with clinically more severe disease such as DMD and SMA compared with BMD and facioscapulohumeral muscular dystrophy.^16,17^ Moreover, its levels seemed to decrease with age in patients with DMD. Thus, downregulation of myostatin may be due to an intrinsic disease process in patients with clinically more severe disease, or it may be a direct consequence of the amount of muscle mass (e.g., the amount of muscle tissue that is able to produce myostatin) or a combination of both.

The results of our study are in line with a previous finding in DMD that higher Cr/Crn is related to lower motor performance measured by functional performance.^15^ Lower myostatin levels correlated weakly with decreased function in patients with DMD and limb-girdle muscular dystrophy type 2B.^16^ These results could be replicated in our BMD cohort when only taking baseline measurements into account for myostatin (ρ = −0.410, *p* = 0.052, data not shown), but not for Cr/Crn (ρ = 0.279, *p* = 0.177, data not shown). In addition, myostatin was significantly lower in nonambulant patients with DMD and BMD compared with patients who were still able to walk.^16^ However, given the progressive nature of diseases such as DMD and BMD, it is difficult to demonstrate a relationship between a biomarker and functional performance in cross-sectional studies, where age alone is often associated with functional decline. In our study, a lower Cr/Crn and higher myostatin levels were related to better functional performance after correction for age, therefore supporting the conclusion of these markers being associated with concurrent performance on top of the effect of age.

Despite the strong correlation of myostatin and Cr/Crn with performance, we observed a moderate and significant association between the biomarkers and the average yearly decline only for the 6MWT, whereas the correlation with the NSAA and TMRv was weak and not significant. The 6MWT was originally developed as a tool to measure the global and integrated response of all systems involved during exercise, such as the pulmonary and cardiovascular systems, and muscle metabolism.^42^ It functions as a measure of endurance, whereas the TMRv measures transient peak activity, and the NSAA is an important measure of daily life activities. Although biomarkers such as Cr/Crn and myostatin reflect change in endurance over the years rather than change in transient peak activity or daily life activities, it is possible that the lack of clear functional decline for part of the patients in any functional measurement in our study (manuscript in preparation) over the 4 studied years does not allow to identify these changes in performance tests. This is especially true for functional scales, such as the NSAA, where floor/ceiling effects were observed, therefore masking the possibility to detect yearly functional changes and therefore the correlation with the biomarkers.

The prediction of concurrent functional performance was more accurate using myostatin and Cr/Crn together with age compared with when a single predictor was used. These results for the prediction of concurrent function are in line with previous studies in which several prognostic prediction models for future changes in function were developed in an effort to optimize trial design in DMD. These models included up to 1,137 patients with 23,305 observations and reported more accurate prediction of future change in functional performance using multiple predictors as opposed to a single predictor. The explained variance of future performance was up to 50%–60% using combined clinical factors such as multiple measures of ambulatory function and steroid use.^43-45^ Despite the smaller number of individuals and observation, our models resulted in a high prediction accuracy, indicating that the addition of creatine, creatinine, and myostatin leads to an improvement in the accuracy of performance prediction. Furthermore, the absence of additive accuracy of dystrophin to the prediction is in line with previous studies from our group and others demonstrating that the dystrophin percentage has a limited correlation with functional performance, although the dystrophin percentage was only available in about half of the patients with BMD in our study.^24,46^ In one other study with 52 patients, dystrophin showed moderate positive correlations with several functional measures.^47^

Several limitations should be acknowledged. Withdrawal of blood was variable in and between patients during the study day, taking place either before or after the clinical assessments. We included only ambulant assessments in the analysis. Dystrophin was only available cross-sectionally in a subset of the patients. This may influence the accuracy of the prediction of concurrent performance. We could not control this study for dietary/supplemental intake and body lean mass. This may affect total biomarker levels and should be investigated in future studies. Although our study includes longitudinal serum samples and clinical data required to identify potential monitoring biomarkers, the presented data show small changes over time for both Cr/Crn and myostatin and functional scales. This currently limits the possibility to more closely define the context of use of these substances.

In conclusion, Cr/Crn and myostatin may be used as monitoring biomarkers in BMD because higher Cr/Crn and lower myostatin were associated with patients' performance after correction for age, and they improved the prediction of concurrent functional performance when combined with age. A longer follow-up, increasing the sample size and/or focusing on patients with a more similar rate of functional decline is needed to get more insight in the longitudinal relationship of Cr/Crn and myostatin with function and disease milestones in BMD to clarify their potential to serve as monitoring biomarkers. In addition, the correlation between the serum biomarkers and quantitative MRI measures should be investigated because it has been shown that muscle MRI may also be used as a biomarker for disease progression in DMD and BMD.^48,49^

## Glossary

6MWT: 6-Minute Walking Test
ADP: adenosine diphosphate
ATP: adenosine triphosphate
BMD: Becker muscular dystrophy
CK: creatine kinase
Cr/Crn: creatine/creatinine_ratio_
CV: coefficient of variation
DMD: Duchenne muscular dystrophy
FVC: forced vital capacity
ICC: intraclass correlation coefficient
NSAA: North Star Ambulatory Assessment
TA: tibialis anterior
TMRv: 10-meter run velocity
